# Supplementary material for: A Paleocene penguin from New Zealand substantiates multiple origins of gigantism in fossil Sphenisciformes
Source: Nat Commun. 2017 Dec 12;8:1927. doi: 10.1038/s41467-017-01959-6 (PMC5727159; doi:10.1038/s41467-017-01959-6)
Supplement: Supplementary file 1 — Supplementary Information [file 41467_2017_1959_MOESM1_ESM.pdf]

Supplementary Information

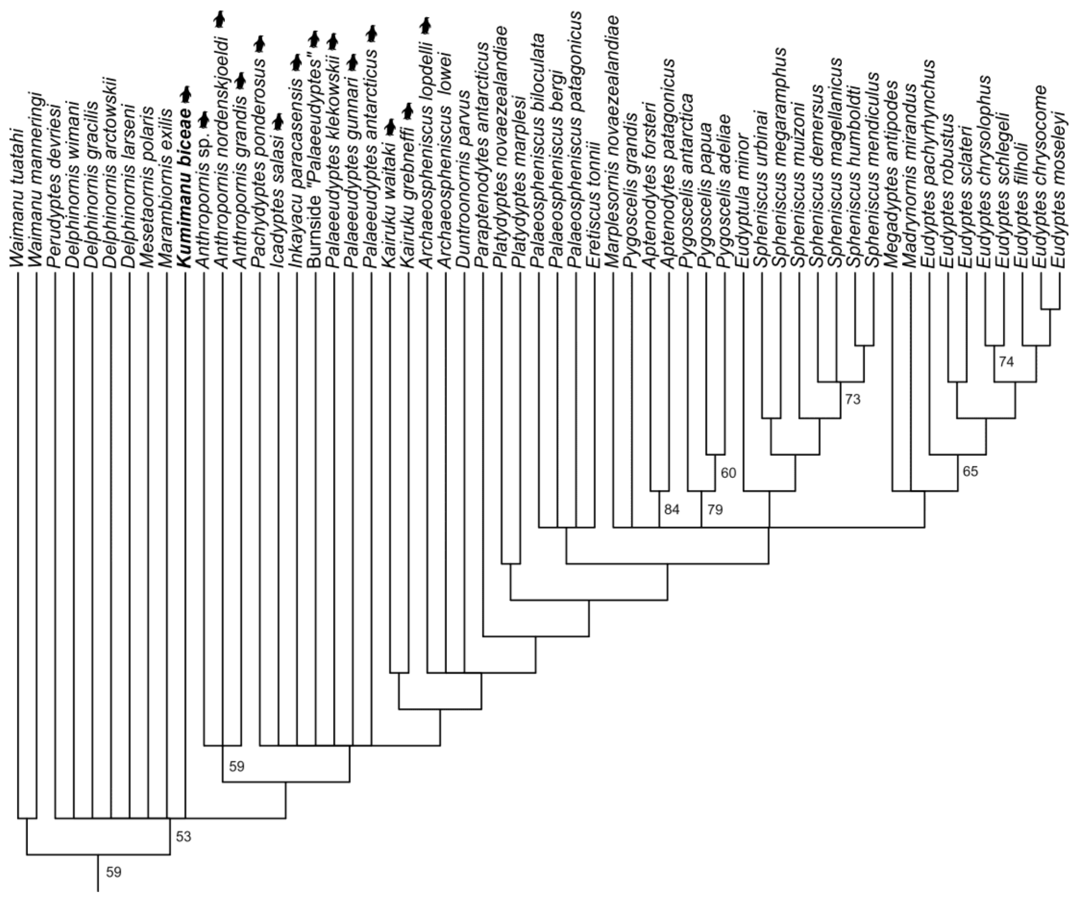

Supplementary Fig. 1 Strict consensus tree of 10000 most parsimonious tree resulting from the phylogenetic analysis of the primary data set with all taxa included (L = 614, CI = 0.58, RI = 0.90). Bootstrap support values (majority rule consensus) are indicated next to the internodes, unsupported nodes were collapsed. The penguin icons denote giant taxa with a size exceeding that of the Emperor Penguin.
