# Supplementary material for: A Paleocene penguin from New Zealand substantiates multiple origins of gigantism in fossil Sphenisciformes
Source: Nat Commun. 2017 Dec 12;8:1927. doi: 10.1038/s41467-017-01959-6 (PMC5727159; doi:10.1038/s41467-017-01959-6)
Supplement: Supplementary file 3 — Description of Additional Supplementary Files [file 41467_2017_1959_MOESM3_ESM.pdf]

File Name: Supplementary Data 1
Description: Nexus file of the full data set (245 characters, 72 taxa).

File Name: Supplementary Data 2
Description: Nexus file of the data set with a reduced taxonomic sampling and two additional characters added (247 characters, 27 taxa).
